# Economy in the Generation and Use of Steam

**Published:** 1914-07-25

**Authors:** 


### ECONOMY IN THE GENERATION AND USE OF STEAM.*

### III.—Prevention of Waste.

In all steam plant the great object to be attained is the use of the largest proportion of the heat liberated by the combustion of the fuel in the boiler furnace. Engineers express this fact by saying that they aim at obtaining the highest possible "efficiency" of their steam plant. There is the efficiency of the boiler, of the steam pipes, of the apparatus which uses the steam, of the condenser when one is used, and so on. Each portion of each apparatus has its own efficiency, and the combination of the efficiencies of the whole of them makes up that of the apparatus itself. Thus, in the boiler there is the efficiency of the furnace, that of the flues, that of the circulation, that of the transmission of heat from the hot gases to the water, and so on. Each portion of each apparatus may and does waste a certain amount of heat. The waste in the boiler furnace, due to the entry of cold air when the furnace doors are open, has already been alluded to. There is another serious source of waste, due to the entry of cold air, that is very often overlooked. With both the Lancashire boiler and the water-tube boiler brickwork is employed to enclose the structure. In the Lancashire boiler—the long, cylindrical boiler that has been so largely used in hospitals—the lower part of the boiler itself rests in a setting of brickwork, separated from the bricks by supports designed for the purpose.

The spaces between the outer shell of the boiler and the brickwork are used as flues. The hot gases, after passing through the internal flues of the boiler, pass through the flues underneath the boiler, and then back by those on each side of the boiler, thence passing either to the "economiser" or directly to the chimney. With old brickwork, or with brickwork which has been badly set, there are often cracks, through which air passes from outside to the flues, and mixes with the hot gases. The air which enters in this way dilutes the hot gases; it abstracts heat from them to raise its own temperature; and so they pass on at a lower temperature than they otherwise would do. The lowered efficiency of many a Lancashire boiler has been traced to this cause.

In the water-tube boiler the water circulates through tubes around which the hot gases play; but the whole thing is built inside brickwork. In the same way, if the brickwork is bad, or if it is neglected, and cracks are allowed to form in it, the hot gases inside will be diluted, and will not perform their proper work.

It may be mentioned also that where cracks of this kind are formed, their tendency is to increase in size; because the friction of the air through them tends to rub away a portion of the brickwork on each side.

Another source of waste of heat is radiation and convection from parts of the boiler itself, and from the steam pipes, if they are exposed to the atmosphere. Lancashire boilers, as explained above, sit in the brickwork which forms the flues; their upper portions are exposed to the atmosphere. As the shell of the boiler is at a very much higher temperature than the surrounding atmosphere, heat is lost directly by radiation from the surface of the boiler shell; and probably to a much greater extent by convection currents. The same thing applies to steam pipes. Necessarily they are at a higher temperature than the surrounding atmosphere; and the same losses of heat take place from their surfaces.

The remedy for this is to cover the exposed surface of the boiler and the surfaces of the steam pipes with one of the non-conducting compositions that are on the market. There are a number of substances whose resistance to the passage of heat is comparatively high. The substance which offers the highest thermal resistance is silicate cotton, or "slag wool," as it is often called. Mica has also been formed into coverings for boilers and steam pipes, and is very efficient. Many substances which are very bad conductors of heat cannot be employed for covering boilers and steam pipes, because while they resist the passage of heat, they do not stand high temperatures; they disintegrate, and in some cases may actually be destroyed. There are a number of patented substances on the market that are used for covering boilers and steam pipes, sold at various prices. One rule can be given in connection with this subject; with any given substance, the greater the thickness, the greater is the resistance offered to the passage of heat through it.

One other hint may be given here. There is an economic law governing the use of thermal insulators. The insulation of boilers and steam pipes, however it may be carried out, costs money; and when the interest on the outlay involved in covering the exposed parts of any steam plant equals the cost of the heat that would be lost by leaving that exposed, no advantage is to be gained by spending more money upon the covering.

* Previous articles on this subject appeared on May 30 and June 13.

